# Quality indicators for pharmaceutical care: a comprehensive set with national scores for Dutch community pharmacies

**DOI:** 10.1007/s11096-016-0301-x

**Published:** 2016-04-23

**Authors:** Martina Teichert, Tim Schoenmakers, Nico Kylstra, Berend Mosk, Marcel L. Bouvy, Frans van de Vaart, Peter A. G. M. De Smet, Michel Wensing

**Affiliations:** Department of IQ Healthcare, Radboud Institute for Health Sciences, Radboud University Medical Centre, PO Box 9101, 6500 HB Nijmegen, The Netherlands; Royal Dutch Pharmacists Association (KNMP), 2514JL The Hague, The Netherlands; Healthcare Inspectorate, Utrecht, The Netherlands; National Health Care Institute, Diemen, The Netherlands; SIR Institute for Pharmacy Practice and Policy, Leiden, The Netherlands; Division of Pharmacoepidemiology and Clinical Pharmacology, Department of Pharmaceutical Sciences, Utrecht University, Utrecht, The Netherlands

**Keywords:** Community pharmacies, Pharmaceutical care, Quality improvement, Quality indicators, The Netherlands

## Abstract

*Background* The quality of pharmaceutical care in community pharmacies in the Netherlands has been assessed annually since 2008. The initial set has been further developed with pharmacists and patient organizations, the healthcare inspectorate, the government and health insurance companies. The set over 2012 was the first set of quality indicators for community pharmacies which was validated and supported by all major stakeholders. The aims of this study were to describe the validated set of quality indicators for community pharmacies and to report their scores over 2012. In subanalyses the score development over 5 years was described for those indicators, that have been surveyed before and remained unchanged. *Methods* Community pharmacists in the Netherlands were invited in 2013 to provide information for the set of 2012. Quality indicators were mapped by categories relevant for pharmaceutical care and defined for structures, processes and dispensing outcomes. Scores for categorically-measured quality indicators were presented as the percentage of pharmacies reporting the presence of a quality aspect. For numerical quality indicators, the mean of all reported scores was expressed. In subanalyses for those indicators that had been questioned previously, scores were collected from earlier measurements for pharmacies providing their scores in 2012. Multilevel analysis was used to assess the consistency of scores within one pharmacy over time by the intra-class correlation coefficient (ICC). *Results* For the set in 2012, 1739 Dutch community pharmacies (88 % of the total) provided information for 66 quality indicators in 10 categories. Indicator scores on the presence of quality structures showed relatively high quality levels. Scores for processes and dispensing outcomes were lower. Subanalyses showed that overall indicators scores improved within pharmacies, but this development differed between pharmacies. *Conclusions* A set of validated quality indicators provided insight into the quality of pharmaceutical care in the Netherlands. The quality of pharmaceutical care improved over time. As of 2012 quality structures were present in at least 80 % of the community pharmacies. Variation in scores on care processes and outcomes between individual pharmacies and over time can initiate future research to better understand and facilitate quality improvement in community pharmacies.

## Impacts on practice

- A validated set of indicators is useful to measure pharmaceutical care in community pharmacies.
- A set of practice indicators is best compiled in cooperation with pharmacists and external stakeholders e.g. healthcare inspectorate, patient organizations and health insurers.
- Measurement of indicators in pharmacy practice and feedback of indicator scores results in overall score improvement.
- Further research is needed to better understand and facilitate quality improvement in individual pharmacies.

## Introduction

Quality indicators in healthcare address measurable aspects of relevant systems, processes and outcomes. They provide insight into the performance of care providers and are used to stimulate continuous improvement of patient care [1]. Various quality indicator sets have been introduced throughout the western world [2–6]. Among others these sets are used to assess and improve the quality of medical practice [7, 8].

To assess pharmaceutical care as the pharmacist’s contribution to the care of individuals in order to optimize medicines use and improve health outcomes [9], in 2008 a national set of quality indicators has been established for community pharmacies in the Netherlands [10]. This was initiated by the Royal Dutch Pharmacists Association (KNMP) and the Netherlands Healthcare Inspectorate. The initial aims of the 2008 indicators were to gain insight into the quality of pharmaceutical services for supervision purposes, and to increase awareness among individual community pharmacists about their own performance. Since 2008, data on specific indicators have been collected from all community pharmacies in the Netherlands. All indicator scores were self-reported by pharmacists. To stimulate internal quality improvement, community pharmacists were annually given feedback reports with their own scores relative to those of the other pharmacists in the Netherlands.

As of 2010 other major stakeholders were involved in the quality measurement of community pharmacies. Beside community pharmacists and the Healthcare Inspectorate, this included representatives of patient and consumer organizations and health insurance companies. These parties were primarily interested in information that enabled patients to make a conscious choice of pharmacy based on performance. Thus beside internal quality assessment the set of quality indicators had to facilitate also external comparisons between pharmacies. Consequently under the supervision of a national steering body and with involvement of all major stakeholders an instrument was developed and used to validate the 2011 set for content validity, absence of selection or measurement bias, and statistical reliability [11]. This set of quality indicators was then thoroughly revised. Information for the resulting 2012 set was requested from all Dutch community pharmacies in 2013.

The comprehensive set of quality indicators for Dutch community pharmacies and it’s results may be useful for other countries and healthcare systems to increase awareness of individual community pharmacists about their own performance, improve supervision by the healthcare inspectorate and enable patients and health insurance companies to differentiate between pharmacies on the basis of performance.

### Aim of the study

This study aimed to present a comprehensive quality indicator set for community pharmacies and to report the scores for these indicators as supplied by the majority of Dutch community pharmacies. In subanalyses the score development over 5 years was described for those indicators, that have been surveyed earlier, and the score consistency within one pharmacy over time was assessed.

### Ethics approval

Data of pharmacists and patients were coded and anonymised prior to analysis. Use of observational data in descriptive retrospective studies in the Netherlands is not considered as an interventional trial according to Directive 2001/20/EC and to Dutch legislation, and therefore does not need to be submitted to a medical ethic committee for approval.

## Methods

### Study design

In this retrospective study information on the validated quality indicator set for the year 2012 was used. This information was requested in 2013 from all 1981 Dutch community pharmacies at that time [12]. Scores were calculated as a cross-sectional measurement of the quality of pharmaceutical care in the Netherlands.

For indicators that were measured as well in the prior 4 years and remained unchanged during the study period, in a longitudinal analysis the development of scores during the whole study period of 5 years was described and the score consistency within one pharmacy during time was assessed.

### Setting for data collection

On average, each pharmacy served around 8000 clients in 2012 [12]. In the Netherlands clients mostly visit one particular community pharmacy [13]. All clients are registered in the pharmacy information system.

Community pharmacists in the Netherlands have a legal and professional responsibility to optimize the safe and effective use of medication, in cooperation with the prescribers. This implies that pharmacists can intervene if a prescribing does not follow the guidelines or does not seem suitable for an individual patient. According to their responsibility to ensure safe and effective medication use, pharmacists generally had high intrinsic motivation to explore their performance in providing pharmaceutical care.

Data have been collected using this national survey for 5 years, and annual participation is a condition for receiving a quality management certificate. The healthcare inspectorate specifically visits any non-responders. The process of data collection was widely announced in professional circles. Although reporting on indicators was voluntary, the relevance of providing data for both internal quality assessment and external accountability was emphasized in all communications [14–17].

### Quality indicator survey tool

All Dutch community pharmacies were invited to complete the online quality indicator survey on 2012 between April and May 2013. This included 66 indicators within ten categories: ‘Quality management’, ‘Continuity of care’, ‘Communication with the patient’, ‘Clinical risk management’, ‘Compounding’, ‘Dispensing’, ‘Follow up of pharmacotherapy guidelines’, ‘Counseling’, ‘Logistics’, and ‘Training of pharmaceutical staff’. Information on each aspect was provided by the responsible pharmacist.

The quality indicators measured the presence of quality systems (‘structures’) and the performance of processes. As information on the clinical consequences of pharmaceutical care for individual patients was not available, outcomes of dispensings for patient groups were used as ‘outcome’ indicators (e.g. the concomitant use of interacting drugs or the absence of concomitant preventive co-medication).

The online survey contained automated controls, for example to prevent reporting of percentages above 100 % and to alert for missing scores. It also provided pharmacists with background information about specific quality indicators when necessary. Pharmacists could also obtain support from technical and professional helpdesks by e-mail or telephone.

### Concurrent source of dispensing outcomes for quality indicator score validation

Information for numerical indicators based on the outcomes from dispensing to individual patients could also be measured by routinely-collected data from the Dutch Foundation of Pharmaceutical Statistics (SFK). The SFK collects drug dispensing data for nearly all community pharmacies [12]. Using validated algorithms, the indicators were calculated for each community pharmacy from the dispensing data delivered to the SFK, and median (50th percentile), 10th and 90th percentiles were available as quality indicator scores from that source. For answering the online survey, pharmacists could use the information from their own pharmacy information system or they could retrieve their indicator scores as pre calculated by the SFK from their dispensing data through a secure website of SFK.

### Data analysis

Scores on the 2012 set were either expressed as categorical variables (yes/no) or as numerical variables (either a number or a proportion). Scores of categorically-measured quality indicators were given as the percentage of pharmacies reporting the presence of a quality aspect. For numerical quality indicators, the mean of the scores was reported, and the variance in answers was expressed as the 5th and 95th percentile.

For the longitudinal analyses, data from the pharmacies responding to the 2012 set were linked to their corresponding scores from the previous 4 years. Ten quality indicators (five on structures, one on process and four on dispensing outcomes) were surveyed in the same way over all five study years. For these quality indicators, the scores were also calculated for the preceding years.

The intra-class correlation coefficient (ICC) was calculated to compare the variance of scores for individual pharmacies over time (indicating changes over time for a particular pharmacy) to the overall variation in scores over the same period. The ICC is an indication of the correlation of scores for a quality indicator reported over time from the same pharmacy. The ICC could be calculated from logistic (for categorical indicators) and linear (for continuous indicators) mixed model analysis [18]. In this analyses, the repeatedly measured scores for an indicator were clustered within individual pharmacies. For continuous variables, the ICC can be calculated by dividing the variance of scores between community pharmacies by the total variance, where the total variance is defined as the sum of the variance between and within pharmacies [18]. For dichotomous variables, the ICC can be calculated with an additional equation [18]. The ICC can take values between 0 and 1. A high ICC (close to 1) means that the scores changed little for an individual pharmacy over time compared with the total variance in scores of all community pharmacies.

Descriptive statistics were performed with PASW statistics 20.0 (IBM Corp., Chicago, IL, USA). Stata SE-2009 (StataCorp LP Statistics/Data Analysis StataCorp, Texas, USA) was used to assess the ICC.

## Results

Information was provided by 1739 of the 1981 Dutch community pharmacies (88 %). Table 1 gives the characteristics of the community pharmacies providing information for the quality indicator set on 2012. On average, each employed 1.3 pharmacists and 6.4 pharmacy technicians. Most pharmacies had a separate room for counseling and participated in night and weekend services. Two-thirds of them cooperated with other healthcare providers in a structured way. One-third also supplied nursing homes. One-third compounded medication within their pharmacy and <5 % also supplied other pharmacies with such preparations.

**Table 1:** Characteristics of Dutch community pharmacies

Mean full time equivalent (38-h working week) pharmacists employed per pharmacy (5th and 95th percentile)	1.36 (0.8; 2.4)
Mean full time equivalents (36-h working week) pharmacy technicians employed per pharmacy (5th and 95th percentile)	6.37 (2; 12)
Percentage of community pharmacies with a separate room available for counselling	96.4
Percentage of community pharmacies that supply nursing homes	31.1
Percentage of community pharmacies that participate in night and weekend services	99.2
Percentage of community pharmacies that cooperate with other pharmacists and healthcare providers for pharmaceutical care in a structured way	70.2
Percentage of community pharmacies that compound medicines within the pharmacy	30.9
Percentage of community pharmacies that supply of compounded medications to more than one other pharmacy	4.1

Based on the answers of 1739 community pharmacies who completed the questionnaire for the quality indicator set in 2012

Table 2 shows the 66 quality indicators across 10 categories, and their scores on the presence of quality structures, the degree to which pharmacies followed recommended procedures and outcomes from dispensing to individual patients. Within the set, 29 indicators measured the presence of structures, 24 focused on processes and 13 covered dispensing outcomes. Indicator scores showed high volumes for the presence of quality structures. For the presence of quality structures for instance, 84 % reported the presence of a validated quality certificate (QI 1.1), and 94 % responded that they had patients’ experiences within the past 3 years evaluated by a professional external party (QI 1.2). The presence of structures providing information about patients’ actual drug use (QI 2.1), contraindications (QI 4.3) and allergic reactions (QI 4.4) were reported by more than 95 % of those responding.

**Table 2:** Scores for the quality indicator set for community pharmacies in 2012

	Score^a^	Type
1. Quality management		
1.1 Presence of a valid quality management certificate^b^	84.4	S
1.2 Evaluating patients’ experiences within the past 3 years^b^	93.9	S
1.3.1 Availability of a procedure for registration of errors (e.g. wrong dosage, wrong substance, wrong compounding) that occurred during the work process in the pharmacy and that were realized *before* the drug reached the patient^b^	73.2	S
1.3.2 Number of registered errors which occurred during the dispensing of medication and that occurred during the work process in the pharmacy and that were realized *afte*r the drug reached the patient^c^	42.2 (0; 205)	P
1.4 Presence of a registration system for errors that occurred during the work process within the pharmacy and that *did* reach the patient^b^	98.8	S
1.5 Number of registered errors that *did* reach the patient^c^	18.0 (0; 64)	P
1.6 Number of registered complaints made by patients^c^	29.1 (0; 150)	P
1.7 Number of registered errors reported to a national registration of errors^c^	1.5 (0; 3)	P
2. Continuity of care		
2.1 Attitude of the pharmacist to obtain information on patients’ actual drug use before dispensing and to register this information in the patients’ record^b^	95.0	S
2.2.1 Participation in pharmacotherapy audit meetings with general practitioners (GPs)^b^	98.7	S
2.2.2 Participation in pharmacotherapy audit meetings on a regular basis and with specific agreements^b^	84.6	S
2.3 Percentage of patients older than 70 years with at least 5 different drug classes in chronic concomitant use, for whom the pharmacist contributed to the exchange of actual drug use information between the general practitioner and the hospital^c^	54.7 (0; 100)	P
2.4 The pharmacy staff always informs the anticoagulation directly in case of dispensing co-trimoxazole to a coumarin user^b^	99.5	S
3. Communication with the patient		
3.1 Percentage of patients with a first dispensing of inhalation medication who had been offered information about its use^c^	70.0 (4; 100)	P
3.2 Percentage of users of inhalation medication with subsequent use of oropharyngeal antimycotics^c^	1.48 (0.5; 16)	O
3.3 Presence of individual education programs and plans for every pharmaceutical staff member^b^	94.6	S
4. Clinical risk management		
4.1 Parameters for clinical risk management in the pharmacy information system are implemented according to prevailing guidelines^b^	98.8	S
4.2 In case of an interaction actions taken are electronically registered	97.9	S
4.3 Availability of protocols for informing on contra indications for all patients, especially for new patients^b^	98.6	S
4.4 Availability of protocols for informing on allergic reactions for all patients, especially for new patients^b^	98.9	S
4.5 Availability of protocols to check on the dosage of active components for compounded medication for children up to 6 years^b^	97.5	S
4.6 Dosage in compounded mediation for children up to 6 years is checked by the pharmacist in at least 80 % of all compounding for children younger than 6 years^b^	83.9	P
4.7 Absolute number of coumarin users with concomitant use of co-trimoxazole^c^	0.69 (0; 2)	O
5. Compounding		
5.1 Availability of written agreements on responsibilities for external compounding on checking the weight of capsules, analytical tests of samples and a final control by a pharmacist^b^	96.5	S
5.2 Availability of a standard operation procedure for the release of compounded medication before dispensing to the patient^b^	98.8	S
5.3.1 Percentage of medication compounded for individual patients for which a standardized procedure was followed^c^	75.4 (6; 100)	P
5.3 2 Percentage of compounding of batches with a validated procedure followed of all batch compounding^c^	87.9 (16; 100)	P
6. Dispensing		
6.1.1 Availability of automated dose dispensing for eligible patients^b^	92.0	S
6.1.2 If automated dose dispensing was used the actual guideline was followed by as well the pharmacist as the supplier^b^	98.9	S
6.2 For weekly dosed trays a system was available to control on drug use as prescribed	97.5	S
7. Follow up of pharmacotherapy guidelines		
7.1.1 Percentage NSAID users >70 years with concomitant gastroprotection^c^	84.7 (70; 96)	O
7.1.2 Action was taken by the pharmacist in at least 80 % of the cases to add gastroprotection to NSAID users >70 years for whom this co-medication was lacking^b^	32.3	P
7.2.1 Percentage of patients using nitrates with concomitant antithrombotic medication^c^	93.0 (86; 100)	O
7.2. Action was taken by the pharmacist in at least 80 % of the cases to add antithrombotic medication to nitrate users for whom this co-medication was lacking^b^	18.0	P
7.3.1 Percentage of patients using opioids with concomitant laxatives^c^	54.1 (35; 76)	O
7.3.2 Action was taken by the pharmacist in at least 80 % of the cases to add laxatives to opioid users in whom this co-medication was lacking^b^	14.3	P
7.4 Percentage of patients under 6 or above 70 years of age with asthma inhalers and an additional inhalation device dispensed during the previous 24 months^c^	69.0 (49; 86)	O
7.5 Percentage of simvastatin as the first statin dispensed^c^	67.0 (34; 93)	O
7.6 Percentage of cardiovascular patients with concomitant statin use^c^	75.7 (68; 83)	O
7.7 Percentage of triptan users without overuse within all triptan users^c^	93.3 (87; 99)	O
7.8 Percentage of first dispensings of hypnotics with an amount for less than 15 days within all first hypnotic dispensings^c^	69.9 (47; 91)	O
7.9 Percentage of proton pump inhibitor (PPI) users with preferred PPIs according to national guidelines within all PPI users^c^	82.2 (71; 91)	O
7.10 Percentage of first dispensings of generic diclofenac, ibuprofen or naproxen within all first NSAID dispensings^c^	84.7 (67; 96)	O
7.11 Percentage of COXib users without co-medication related to ischemic cardiovascular diseases within all COXib users^c^	83.4 (73; 94)	O
7.12.1 The pharmacist followed additional courses for the performance of Medication Reviews^b^	73.8	P
7.12.2 Medication Reviews are performed according to the professional guideline in cooperation with GPs and patients^b^	92.6	S
7.12.3 Performance of at least 20 Medication Reviews according to the professional guideline in cooperation with GPs and patients^b^	57.3	P
8. OTC counseling		
8.1 Medication surveillance is conducted according to professional protocols^b^	99.6	S
8.2.1 Percentage of filled protocols for patient counseling within first dispensing of orlistat^c^	76.9 (0; 100)	P
8.2.2 Percentage of filled protocols for patient counseling within first dispensing of dextromethorphan^c^	66.9 (0; 100)	P
8.2.3 Percentage of filled protocols for patient counseling within first dispensing of hypericum^c^	64.6 (0; 100)	P
8.2.4 Percentage of filled protocols for patient counseling within first dispensing of domperidon^c^	70.5 (0; 100)	P
8.2.5 Percentage of filled protocols for patient counseling within first dispensing of hydrokinin^c^	67.5 (0; 100)	P
9. Logistics		
9.1 Suppliers of compounding material were assessed according to the professional guideline^b^	99.4	S
9.2 Percentage of suppliers for compounding or package material that were assessed for their reliability as stated by the guideline for reliable supplier s^c^	74.6 (13;100)	S
9.3 Availability of a valid system to check on expired drugs^b^	99.7	S
9.4 Official drug recalls were performed^b^	99.8	S
9.5.1 Number of relevant recalls received in calendar in question^c^	8.0 (0;15)	P
9.5.2 Number of not completely finished recalls^c^	0.9 (0; 8)	P
9.5.3 Not completed drug recalls were due to a too high effort to address patients^b^	3.7	P
9.6.1 Number of internally reported expired medication before the drug was dispensed^c^	1.8 (0; 7)	P
9.6.2 Number of dispensed expired medication that was reported by the patient and thus was noticed after dispensing^c^	1.8 (0; 2)	P
10. Training of pharmaceutical staff		
10.1.1 Percentage of pharmaceutical staff with a personal development plan^c^	60.0 (0; 100)	S
10.1.2 Percentage of pharmacy technicians who were registered in a central quality registration system for education^c^	33.4 (0; 100)	S
10.2 Participation in a national program for patient reported side effects drugs of the national pharmacovigilance center^b^	87.5	S
10.3 Number of patient reported side effects announced to the national pharmacovigilance center^c^	1.4 (0; 5)	P
10.4 Percentage of employees involved in pharmaceutical care that followed an education in communication skills^c^	32.2 (0; 100)	S

Based on the answers of 1739 community pharmacies

S, system indicator; P, process indicator; O, outcome of dispensing indicator

^a^For numerical quality indicators, scores for the 5th and 95th percentiles are given in brackets

^b^Categorical indicators are given as percentage of community pharmacies answering ‘yes’

^c^Numerical quality indicators are given as the mean of the absolute numbers or percentages given as answers

Scores were lower for processes and dispensing outcomes, and the intervals between the 5th and 95th percentile were broad for indicators measured on a numerical scale. For instance, the process indicator ‘Percentage of patients older than 70 years with at least five different drug classes in chronic concomitant use, for whom the pharmacist contributed to the exchange of actual drug use information between the general practitioner and the hospital’ (QI 2.3) had a mean of 55 % with a range between the 5th and 95th percentile between 0 and 100 %. The outcome of dispensing indicator ‘Percentage of patients using opioids with concomitant laxatives’ (QI 7.3.1) had a mean of 54 % with a range between the 5th and 95th percentile from 35 to 75 %.

For longitudinal subanalyses on the development of scores during time and trends in score development of individual pharmacies, ten quality indicators were available (Table 3). Overall, the scores for these indicators improved from 2008 to 2012. As an example for the score development of the categorical indicator, ‘Participation in pharmacotherapy audit meetings on a regular basis and with specific agreements’ improved from 78 % in 2008 to 85 % in 2012 (QI 2.2.2). An example for a categorical indicator is the ‘Mean percentage of patients with a first dispensing of inhalation medication who had been offered information about its use’ (QI 3.1). This score increased from 58 % in 2008 to 70 % in 2012 Scores of those quality indicators, which were measured repeatedly, but not in all years, generally also improved (data not shown). The ICCs for the quality indicators had a range between 0.01 for ‘number of coumarin users with concomitant use of co-trimoxazole’ (QI 4.7) and 0.90 for the ‘presence of a valid quality management certificate’ (QI 1.1) (Table 2).

**Table 3:** Trends in quality indicator scores over 5 years of measurement

	Type	2008	2009	2010	2011	2012	ICC^a^
*Categorical quality indicators (percentage of community pharmacies with a positive answer within all given answers)*							
1.1 Presence of a valid quality management certificate	S	*63.3*	*67.6*	*75.2*	*83.0*	*84.4*	*0.90*
1.2 Evaluation of patients’ experiences within the past three years	S	*86.7*	*81.6*	*84.4*	*92.8*	*93.9*	*0.48*
2.2.2 Participation in pharmacotherapy audit meetings on a regular basis and with specific agreements	S	*77.7*	*78.8*	*80.6*	*86.2*	*84.6*	*0.18*
4.3 Availability of protocols for informing on contra indications for all patients, especially new ones	S	74.3	89.7	91.5	96.3	98.6	0.89
7.12.3 Medication Reviews are performed according to the professional guideline in cooperation with GPs and patients	S	*20.3*	*26.3*	*39.6*	*50.3*	*57.3*	*0.14*
*Numerical quality indicators (mean of numbers of percentages)*							
3.1 Percentage of patients with a first dispensing of inhalation medication who had been offered information about its use	P	57.9	68.5	72.9	67.4	70.0	0.45
4.7 Absolute number of coumarin users with concomitant use of co-trimoxazole	O	18.2	1.12	0.75	0.71	0.69	0.01
7.1.1 Percentage NSAID users >70 years with concomitant gastro protection	O	70.8	76.8	81.8	84.0	84.7	0.54
7.2.1 Percentage of patients using nitrates with concomitant antithrombotic medication	O	75.8	84.9	90.9	92.0	93.0	0.46
7.3.1 Percentage of patients using opioids with concomitant laxatives	O	44.5	52.6	52.8	56.1	54.1	0.51

Based on data from 1739 community pharmacies (88 % of all Dutch community pharmacies in 2012)

S, presence system indicator; P, process indicator; O, outcome of dispensings indicator

^a^ICC, Intra Class Coefficient, reflecting score variance within pharmacies compared with the total score variance

Comparison of the self reported indicator scores to the scores measured by the SFK as an independent third party from routinely-collected dispensing data showed a high agreement between the two sources “Appendix” section.

## Discussion

A comprehensive set of 66 quality indicators across 10 categories was developed by pharmacists and major stakeholders to continuously improve pharmaceutical care and to compare indicator scores between community pharmacies. Existing quality sets have been developed for specific diseases, such as urinary tract infections [19,] vulnerable patients such as children and older people, and specific settings such as nursing homes [20–26]. Other indicators on the quality of drug prescribing and dispensing mostly focused on drug use patterns [26–28] or described to what extent specific guidelines and recommendations have been followed [28–30].

The scores on this set of validated quality indicators supplied by 88 % of all community pharmacies provided insight into the pharmaceutical care performance of community pharmacies in the Netherlands. All indicators in the set assessed for 2012 and measured in the four preceding years showed a general improvement from 2008 to 2012. Small to moderate effects on professional practice have been previously described for audit and feedback [31]. A Cochrane review on the effects of audit and feedback reported a median change of 1.3 % in numeric outcomes, with an interquartile range of 1–29 % [32]. The changes in the indicator scores in our study were consistent with these results.

Structures to facilitate pharmaceutical care processes and dispensing outcomes for individual patients were present in at least 80 % of all Dutch community pharmacy. In this the presence of a valid quality certificate is the most meaningful, as this was acknowledged from a third, authorized party by national standards [33].

Scores for indicators for processes and dispensing outcomes were between 50 and 90 %. In principle, scores below 100 % suggest potential for improvement. Full compliance with treatment guidelines for all patients at risk is, however, unrealistic, and levels may also differ across indicators. For instance, the indicator ‘Percentage of patients using nitrates with concomitant antithrombotic medication’ (QI 7.2.1) had a score of 93 % in 2012, which had improved continuously from 76 % in 2008. Possibly this is the highest score to achieve in clinical practice for a patient population. The mean percentage of patients on opioids with concomitant use of laxatives (QI 7.3.1) remained between 45 and 56 % without a clear trend over time. The reasons for the deficient implementation of this recommendation should be studied in clinical practice.

Confidence intervals for the numerical indicators showed considerable variation and suggested differences in the performance of individual community pharmacies. Substantial changes in the scores of individual pharmacies were seen over time for users with the unfavorable combination of coumarin with co-trimoxazole (QI 4.7): in 2008, pharmacies had on average 18 coumarin users co-medicating with co-trimoxazole, but this had decreased dramatically by 2012, to an average of less than one per pharmacy. The low ICC of 0.01 shows that the absolute numbers reported by individual pharmacies for this interaction varied considerable over time, probably due to the huge decrease in numbers between 2008 and 2009 within all pharmacies. As the interaction of coumarin with co-trimoxazole should be avoided, this decrease means a substantial improvement in dispensing outcomes for patients [34]. This improvement might be due to increased awareness among community pharmacies resulting from the feedback reports on the first indicator assessment. It might also be due to extra alertness for this interaction by prescribers due to the new recommendations from literature [34].

The scores for the other quality indicators on the outcomes of dispensing processes (QI 7.11, QI 7.21, QI 7.3.1) measured percentages of patients co-medicated according to the guidelines. For these indicators, at least some consistency in the scores reported by individual pharmacies over time was suggested by ICCs between 0.46 and 0.54.The ICCs showed stable scores over time within individual pharmacies for the presence of systems such as a quality management certificate and the availability of protocols to provide information about contraindications (ICC for both aspects was 0.9).Further research should examine what factors might contribute to improvements in individual pharmacies and why some indicator scores improved more than others.

### Strengths and limitations

A key strength of our study was that information was available from 88 % of all Dutch community pharmacies. Sub-analysis on the 12 % of non-responders in 2012 for their scores reported in previous years, however, showed lower scores on all quality indicators than the scores of the pharmacies included in our study. The scores presented here might therefore tend to overestimate the quality of care for community pharmacies in the Netherlands to some extent.

As all quality indicator scores were self-reported by community pharmacists, some of the indicators, especially on the presence of structures and processes, are too easy to claim without actually performing in the desired way. This attitude might be furthermore influenced by an increasing awareness of quality resulting from pay-for-performance policies of health insurance companies could have caused a bias in the scores towards the desired outcomes. Bias from inappropriate reporting of quality indicator scores was also assumed in the measurements of GP scores in the UK [35]. We therefore compared those scores that could be checked with scores calculated in a uniform way by a third party from routinely-collected dispensing data of SFK. These indicators on the outcomes of dispensing could thus not be claimed from self assessment by the pharmacists. The scores from the two sources were in agreement and there was no evidence of structural higher reported scores from self-assessment “Appendix” section. We therefore consider it unlikely that reporting bias influenced the scores presented here.

## Conclusion

A set of quality indicators provided insight into the quality of pharmaceutical care at a national level for pharmacists, health care inspectorate, health insurance companies and patient organizations. Especially the presence of quality structures improved, and as of 2012 they were present in at least 80 % of the community pharmacies. Scores on care processes and on outcomes of dispensing varied between individual pharmacies and over time. These findings can be used in future research to understand the reasons for differences in quality improvement between individual pharmacies.

## Appendix

See Table 4.

**Table 4:** Concurrent source for dispensing outcomes and external validation of indicator scores

Quality indicator	Self-reported scores, median (10th; 90th percentile)^a^	Scores calculated by SFK^b^, median (10th; 90th percentile)^c^
7.1.1 Percentage NSAID users >70 years with concomitant gastro- protection	86.4 (75; 94)	88.7 (77; 95)
7.2.1 Percentage of patients using nitrates with concomitant antithrombotic medication	94.3 (89; 98)	93.6 (88; 98)
7.3.1 Percentage of patients using opioids with concomitant laxatives	53.7 (40; 70)	55.8 (40; 70)
7.4 Percentage of patients under 6 or above 70 years of age with asthma inhalers and an additional inhalation device dispensed during the previous 24 months	71.0 (55; 83)	69.1 (56; 80)
7.5 Percentage of simvastatin as the first statin dispensed	68.6 (43; 89)	62.5 (36; 86)
7.6 Percentage of cardiovascular patients with concomitant statin use	76.3 (70; 82)	74.8 (68; 80)
7.7 Percentage of triptan users without overuse within all triptan users	93.5 (89; 98)	93.3 (89; 98)
7.8 Percentage of first dispensings of hypnotics with an amount for less than 15 days within all first hypnotic dispensings	70.2 (52; 87)	71.2 (54; 87)
7.9 Percentage of proton pump inhibitor (PPI) users with preferred PPIs according to national guidelines within all PPI users	83.4 (74; 90)	83.0 (73; 89)
7.10 Percentage of first dispensings of generic diclofenac, ibuprofen or naproxen within all first NSAID dispensings	86.3 (72; 95)	84.6 (70; 94)
7.11 Percentage of COXib users without co-medication related to ischemic cardiovascular diseases within all COXib users	84.3 (76; 91)	84.1 (76; 92)

^a^Based on the self report of 1739 community pharmacies (88 % of the total)

^b^SFK, Stichting Farmaceutische Kengetallen, Dutch foundation of pharmaceutical statistics

^c^Based on routinely-collected dispensing data from 1882 community pharmacies (95 % of the total)

NSAIDs, non-steroidalantirheumatic drugs; COXib, cyclo oxigenase inhibitor

## References

[1] Campbell SM, Braspenning J, Hutchinson A, Marshall M (2003). Research methods used in developing and applying qyality indicators in primary care. BMJ..

[2] National Institute for Health and Clinical Excellence. How we develop the NICE indicator menu for the QOF. https://www.nice.org.uk/standards-and-indicators/how-we-develop-qof (2009). Accessed 21 April 2016.

[3] Campbell S, Kontopantelis E, Hannon K (2011). Framework and indicator testing protocol for developing and piloting quality indicators for the UK quality and outcomes framework. BMC Fam Pract.

[4] AQUA—Institut für angewandte Qualitätsförderung und Forschung im Gesundheitswesen GmbH. Allgemeine Methoden im Rahmen der sektorenübergreifenden Qualitätssicherung im Gesundheitswesen nach §137a SGB V Version 2.0. General methods for cross-sectoral quality assurance in health care in accordance with §137a Volume V of the Social Insurance Code. http://www.aqua-institut.de/aqua/upload/CONTENT/Projekte/137a/Methodenpapier/AQUA_AllgemeineMethoden_Version_2-0.pdf (2010). Accessed 7 May 2012.

[5] Szecsenyi J, Broge B, Eckhardt J, Heller G, Kaufmann-Kolle P, Wensing M. Tearing down walls: opening the border between hospital and ambulatory care for quality improvement in Germany. Int J Qual Health Care. 2012;24(2):101–4. 10.1093/intqhc/mzr086 PMC3297368 22269341

[6] Romano P, Mull H, Rivard P (2009). Validity of selected AHRQ patient safety indicators based on VA National Surgical Quality Improvement Program data. Health Serv Res.

[7] van Doorn-Klomberg A, Braspenning J, Wolters R, Bouma M (2014). M. W. Effect of accreditation on the quality of chronic disease management: a comparative observational study. BMC Fam Pract.

[8] Elwyn G, Bekkers M-J, Tapp L, Edwards A, Newcombe R, Eriksson T, et al. Facilitating organisational development using a group-based formative assessment and benchmarking method: design and implementation of the International Family Practice Maturity Matrix. Qual Saf Health Care. 2010;19(6):e48. doi:10.1136/qshc.2009.037580. 10.1136/qshc.2009.037580 20511595

[9] Pharmaceutical Care Network Europe P. Position paper on the definition of pharmaceutical care 2013. http://www.pcne.org//upload/files/3_PCNE_Definition_Position_Paper_final.pdf (2013). Accessed 16 Jan 2016.

[10] De Bie J, Kijlstra N, Daemen B, Bouvy M (2011). The development of quality indicators for community pharmacy care. BMJ Qual Saf.

[11] Schoenmakers T, Teichert M, Braspenning J, Vunderink L, de Smet P, Wensing M (2015). Evaluation of quality indiators for Dutch community pharmacies using a comprehensive assessment framework. J Manag Care Pharm.

[12] Dutch Foundation of Pharmaceutical Statistics S. Data en Feiten. http://www.sfk.nl/nieuws-publicaties/data-en-feiten/SFKDataenfeiten2014.pdf (2014). Visited on 17 Jan 2016.

[13] Buurma H, Bouvy ML, De Smet PAGM, Floor-Schreudering A, Leufkens HGM, Egberts ACG (2008). Prevalence and determinants of pharmacy shopping behaviour. J Clin Pharm Ther.

[14] Royal Dutch Pharmacists Association. KNMP maakt het zo makkelijk mogelijk. Kwaliteitsmeting staat weer voor de deur Pharmaceutisch Weekblad. http://www.pw.nl/archief/2010/nummer-02-jaar-2010/2010pw02p35.pdf/view (2010). Accessed 17 Jan 2016.

[15] Teichert M, Van de Vaart F. Kwaliteitsindicatoren worden steeds beter. Pharmaceutisch Weekblad. 14. http://www.pw.nl/archief/2010/nummer-14-jaar-2010/2010pw14p33.pdf/view (2010). Accessed 17 Jan 2016.

[16] Royal Dutch Pharmacists Association. Kwaliteitsmonitor massaal ingevuld Pharmaceutisch Weekblad. http://www.pw.nl/rubrieken/knmp/respons-van-95 (2011). Accessed 17 Jan 2016.

[17] Royal Dutch Pharmacists Association. Extra zorgvuldigheid bij kwaliteitsindicatoren. Pharmaceutisch Weekblad. http://www.pw.nl/rubrieken/knmp/extra-zorgvuldigheid-bij-kwaliteitsindicatoren (2012). Accessed 17 Jan 2016.

[18] Twisk J. Applied multilevel analysis. Cambridge University Press; 2006. p. 46. ISBN 0-521-61498-8:14.

[19] Hermanides HS, Hulscher MEJL, Schouten JA, Prins JM, Geerlings SE (2008). Development of quality indicators for the antibiotic treatment of complicated urinary tract infections: a first step to measure and improve care. Clin Infect Dis.

[20] Campbell S, Cantrill J, Roberts D (2000). Prescribing indicators for UK general practice: Delphi consultation study. BMJ..

[21] Morris C, Cantrill J, Hepler C, Noyce P (2002). Preventing drug-related morbidity–determining valid indicators. Int J Qual Health Care.

[22] Morris C, Cantrill J (2003). Preventing drug-related morbidity–the development of quality indicators. J Clin Pharm Ther.

[23] To T, Guttmann A, Lougheed M, Gershon A, Dell S, Stanbrook M (2010). Evidence-based performance indicators of primary care for asthma: a modified RAND Appropriateness Method. Int J Qual Health Care.

[24] Uphoff E, Wennekes L, Punt C, Grol R, Wollersheim H, Hermens R (2012). Development of generic quality indicators for patient-centered cancer care by using a RAND modified Delphi method. Cancer Nurs.

[25] Caughey GE, Ellett LMK, Wong TY (2014). Development of evidence-based Australian medication-related indicators of potentially preventable hospitalisations: a modified RAND appropriateness method. BMJ.

[26] Martirosyan L, Braspenning J, Denig P, de Grauw W, Bouma M, Storms F (2008). Prescribing quality indicators of type 2 diabetes mellitus ambulatory care. Qual Saf Health Care.

[27] Yoon D, Park I, Schuemie M, Park M, Kim J, Park R (2013). A quantitative method for assessment of prescribing patterns using electronic health records. PLoS One.

[28] Zomerdijk IM, Sayed-Tabatabaei FA, Trifirò G, Blackburn SC, Sturkenboom MC, Straus SM (2012). Risk minimization activities of centrally authorized products in the EU: A descriptive study. Drug Saf.

[29] Malo S, Bjerrum L, Feja C, Lallana M, Moliner J, Rabanaque M (2014). Compliance with recommendations on outpatient antibiotic prescribing for respiratory tract infections: the case of Spain. Basic Clin Pharmacol Toxicol.

[30] Warlé-van Herwaarden M, Valkhoff V, Teichert M, Koffeman A, ‘t Jong G, Sturkenboom M (2014). Development and application of indicators for the reduction of potentially preventable hospital admissions related to medications. Expert Opin Drug Saf..

[31] Jamtvedt G, Young J, Kristoffersen D, O’Brian M, Oxman A (2006). Does telling people what they have been doing change what they do? A systemati review of the effects of audit and feedback. Qual Saf Health Care..

[32] Ivers N, Jamtvedt G, Flottorp S, Young J, Odgaard-Jensen J, French S, et al. Audit and feedback: effects on professional practice and patient outcomes. Cochrane Summaries. http://summaries.cochrane.org/CD000259/audit-and-feedback-effects-on-professional-practice-and-patient-outcomes (2012). Visited 30 Mar 2013. 10.1002/14651858.CD000259.pub3 PMC11338587 22696318

[33] Harmonisatie Kwaliteitsbeoordeling in de Zorgsector. HKZ certificate. http://www.careforlevel.nl/hkz-stichting-harmonisatie-kwaliteitsbeoordeling-in-de-zorgsector/. NA. Accessed 7 Nov 2015.

[34] Schalekamp T, van Geest-Daalderop J, Kramer M, van Holten-Verzantvoort A, de Boer A (2007). Coumarin anticoagulants and co-trimoxazole: avoid the combination rather than manage the interaction. Eur J Clin Pharmacol.

[35] Doran T, Fullwood C, Gravelle H, Reeves D, Kontopantelis E, Hiroeh U (2006). Pay-for-performance programs in family practices in the United Kingdom. N Engl J Med.

